# South Africa

**Published:** 1896-10

**Authors:** 


*Cape Colony.* A bill is under consideration by the Society for Prevention of Cruelty to Animals to have the Parliament pass a law preventing dehorning of cattle except by a qualified veterinarian, or persons holding certificates from the latter or a resident magistrate. Calves may be done by the owners by the application of caustic potash.

Rinderpest threatens very great disaster to the cattle industry of South Africa unless more radical measures are entered upon for its total extermination.

---

Tuberculosis is receiving a great deal of consideration at the hands of the profession in England, and the relationship of bovine and human extension of the disease is receiving a great deal of thought, with much difference of opinion as to the best methods of control.

A recent experiment with the x-rays demonstrated that an ankylosis of the elbow-joint in a man was due to tubercular degeneration. X-rays, consisting of six five-minute applications, were also applied for treatment, after which the patient was able to relax the joint from 15° to an angle of 70°, with a marked diminution of the swelling, pain, and other symptoms. An equally successful result was obtained in the tubercular bursæ of the wrist; five five-minute applications were employed.

